# Occupational pesticide exposure and safety assessment among farmers in Hohoe municipality: An ethnographic qualitative study

**DOI:** 10.1371/journal.pone.0337693

**Published:** 2025-12-02

**Authors:** Forgive Awo Norvivor, Elijah Kwasi Peprah, Doreen Danso, Obed Woani Konutse

**Affiliations:** 1 Department of Epidemiology and Biostatistics, Fred N. Binka School of Public Health, University of Health and Allied Sciences, Hohoe, Ghana; 2 Department of Environmental Health and Sanitation, School of Hygiene, Accra, Ghana; Canadian University Dubai, UNITED ARAB EMIRATES

## Abstract

**Introduction:**

Occupational pesticide exposure poses significant health risks, particularly among smallholder farmers in developing countries like Ghana, where such exposures are common due to widespread subsistence agricultural activities. The Environmental Protection Agency (EPA) of Ghana is responsible for registering and monitoring pesticide use and regulating the presence of unregistered or banned products on the local market; however, this regulation is ineffective. Farmers frequently acquire pesticides directly from vendors who may not possess sufficient training, and the lack of stringent measures facilitates the accessibility of hazardous products. Furthermore, while regulations mandate safe handling and disposal practices, there are also possible oversights at the community level, leaving farmers largely dependent on their own knowledge, practices, or what they have learnt from other colleagues. This study explores the safety practices, perceived exposure levels, and awareness among rural farmers in the Hohoe municipality of Ghana.

**Methods:**

A qualitative ethnographic approach was adopted, involving in-depth interviews with 13 purposively selected farmers with over five years of farming experience. Data was collected using semi-structured interviews guide informed by prior literature. Thematic analysis was conducted using ATLAS.ti version 25, with open and selective coding techniques.

**Results:**

Four major themes emerged: knowledge and practices of pesticide use, health risks and exposure, safety practices, pesticide storage and disposal. Most farmers (approximately 10 out of 13) reported using Glyphosate as their primary herbicide, followed by Paraquat Dichloride and 2,4-D, relying on vendor recommendations for application methods. Common health complaints included eye and skin irritation, waist pain, and temporary vision loss. While some farmers used PPE like boots and gloves, many lacked complete protective gear, especially eye protection. Pesticide containers were often stored at home or discarded on farms or by burning, indicating poor disposal practices. Awareness of pesticide expiry dates and proper dosage was inconsistent.

**Conclusion:**

The study reveals substantial gaps in pesticide safety knowledge and practices among rural farmers, posing significant public health risks. Targeted interventions, including regular training on safe pesticide handling, PPE use, and environmentally sound disposal methods, are critical to reducing occupational exposure and its associated health burdens.

## Introduction

Pesticide poisoning is any illness or health effect resulting from suspected or confirmed exposure to a pesticide within 48 hours [1]; this has been regarded as a serious public health problem among farmers. Although occupational exposure seems to be well-understood problem, it is probably the most common source of exposure that leads to involuntary acute poisoning [2].

Globally, the consumption of pesticides has increased dramatically in recent decades, with the global pesticide market estimated to reach $81.1 billion by 2024, growing at a compound annual growth rate of 4.9% between 2019 and 2024 [3]. In developing countries, including Ghana, the increased use of pesticides in agriculture has been driven by the need to improve crop yields, protection against pests and diseases, and to meet the growing demand for food [4]. However, the safe and responsible use of pesticides remains a significant challenge, especially among smallholder farmers who often have limited access to proper training, protective equipment, and disposal facilities [5].

According to a review by [6], 740,000 cases of Unintended Acute Pesticide Poisoning (UAPP) were reported annually on a global scale. Despite the rising global use of pesticides, comprehensive documentation of pesticide intoxication has only become available in recent decades, which suggests that the true burden may be underestimated. Acute Pesticide Poisoning is particularly concerning, as it not only signals immediate toxic exposure but may also indicate risks of long-term health consequences if exposures are repeated or unmanaged. Beyond acute poisoning, chronic pesticide exposures pose a significant but often underrecognised public health concern. Long-term, low-dose contact, common among farmers and applicators, has been linked to a range of adverse outcomes, including neurotoxicity, endocrine disruption, respiratory impairment, increased risks of cancers and reproductive disorders [7–9]. Such effects often develop insidiously after repeated exposure to organophosphates, carbamates, and glyphosate-based herbicides. Chronic exposure may also exacerbate oxidative stress and immune dysfunction, contributing to progressive systemic toxicity. Therefore, both acute and chronic pesticide exposures must be considered when assessing the overall burden of pesticide-related diseases among agricultural workers, particularly in low- and middle-income countries where regulatory, enforcement, and health surveillance remain limited.

Beyond fatalities, acute poisoning episodes often result in a range of short-term health effects, including headaches, dizziness, nausea, eye irritation, and skin problems that reduce quality of life and productivity. In many cases, these acute symptoms may progress into chronic health issues, thereby compounding the socioeconomic burden on farming households.

The global distribution of Unintentional Acute Pesticide Poisoning (UAPP) estimated that around 385 million cases of pesticide poisoning occur annually, with approximately 11,000 fatalities [10]. This highlighted that nearly 44% of farmers worldwide experience some form of pesticide poisoning each year, underscoring the scale of occupational exposure in agricultural communities.

While the majority of these cases are unintentional, the widespread availability and misuse of pesticides have also contributed to intentional ingestion in some countries. For example, pesticide self-poisoning accounts for a large proportion of suicides in parts of Asia, including China [11]. This dual burden of unintentional and intentional pesticide exposure demonstrates both the occupational hazards faced by farmers and the broader public health risks associated with weak pesticide regulation and control.

This situation is not entirely different in Africa, where the incidence of pesticide poisoning is due to a significant lack of reporting, suggesting that the burden of diseases caused by UAPP is high but frequently underestimated [10]. [12] further reiterated that there was a general lack of attention to acute toxic exposure, particularly acute non-lethal occupational toxic exposure, which may have hampered the development of measures to prevent such toxic exposure at the national and international levels.

This is underscored by studies in developing countries highlighting farmers knowledge and practices being at low to moderate levels about pesticides [11]. These included non-use of Personal Protective Equipment (PPE), unsafe pesticide storage at homes, poor disposal of empty pesticide containers, misuse of pesticides, and relatively low knowledge about pesticide safety labels [13]. Socio-cultural background, risk perceptions, and working conditions exacerbate these occurrences.

While in Africa, [14] elaborated that there are multiple reported instances of pesticide misuse; little research has been done to determine which measures can effectively minimize acute pesticide exposure. Meanwhile, medical care is potentially hindered because people who have acute pesticide poisoning may choose not to seek medical attention for a variety of reasons, including lack of health insurance, language and cultural barriers, inability to take time off work, or lack of access to transportation or medical facilities [15].

The Environmental Protection Agency (EPA) regulates pesticides in Ghana, and agricultural extension officers are responsible for educating farmers on safe handling practices. However, limited enforcement and irregular extension services mean that many farmers depend on agrochemical vendors for advice, which often leads to unsafe behaviours. Studies report misuse, such as applying higher-than-recommended doses, using banned or expired products, and engaging in hazardous practices like reusing containers for household purposes, mixing pesticides with bare hands, and even tasting them [16,17].

Recent observations in rural areas, including the Hohoe municipality, show continued misapplication of agrochemicals and poor adherence to safety protocols [18].

Although pesticide exposure has been widely documented, there is still a lack of in-depth understanding of how farmers in rural Ghana perceive and practice pesticide safety. Most available studies emphasize prevalence rates of poisoning or levels of knowledge, but they often overlook the everyday lived experiences, decision-making processes, and coping mechanisms of farmers who are directly exposed. This creates a critical gap in understanding the cultural, social, and environmental contexts that influence unsafe behaviours such as improper storage, inadequate use of Personal Protective Equipment (PPE), and unsafe disposal practices. This study is designed to address these gaps by providing qualitative insights into how farmers in the Hohoe municipality use and manage pesticides, the health risks they encounter, and the protective strategies they adopt. The findings from this research are crucial for several reasons. First, this study will provide baseline data on occupational pesticide exposure in rural Ghana, where official records are often limited or absent. Also, by capturing farmers’ voices and lived experiences, the study highlights the human dimension of pesticide use, which is often missed in purely quantitative surveys. Moreover, the results can inform tailored health education campaigns, strengthen agricultural extension services, and guide policymakers in developing safer pesticide regulations and sustainable farming practices. Ultimately, the study contributes to protecting the health and livelihoods of farmers, their families, and their communities while promoting environmental safety. Therefore, this study aims to assess occupational pesticide exposure and safety assessment among farmers in the Hohoe Municipality.

## Methods

### Study area

The Hohoe Municipality is located in the Volta Region of Ghana, within the forest–savannah transitional ecological zone. It covers approximately 1,172 square kilometers and shares boundaries with Jasikan, Afadzato South, Kpando, and the Republic of Togo. According to the 2021 Population and Housing Census, the municipality has a population of about 200,000 , with agriculture serving as the dominant livelihood. Over 70% of residents engage in small-scale farming, cultivating crops such as maize, cassava, yams, rice, plantain, cocoa, and vegetables [19]. Hohoe was purposively selected for this study because of its intensive pesticide-dependent farming activities, limited access to agricultural extension services, and socioeconomic vulnerability of smallholder farmers, which make it a high-risk area for occupational pesticide exposure.

### Study design

The study employed a focused ethnographic design, which is well-suited for exploring specific practices and knowledge systems within defined communities over a limited time frame. Unlike classical ethnography that requires prolonged field residence, focused ethnography uses intensive interviewing and field engagement to gain insider perspectives on a particular issue; in this case, farmers’ knowledge and practices surrounding pesticide handling. The approach was chosen to capture culturally embedded practices, language, and meanings around pesticide use in Hohoe. The researcher engaged with participants both in farm and community settings, supplemented interviews with field notes, and maintained reflexive memos to document contextual insights. Qualitative in-depth interviews were conducted with these farmers. The study was conducted and reported in accordance with the RATS (Relevance, Appropriateness, Transparency, Soundness) guidelines for qualitative research [20].

### Sampling procedure

In total, 18 eligible farmers were approached and were expected to participate in the study; however, only 13 consented and participated, yielding a response rate of 72%. The inclusion criteria were farmers with more than five years of farming experience who were actively engaged in pesticide use during crop production. Variation in pesticide application practices (e.g., use patterns, storage methods, PPE adherence, and disposal habits) was sought to enrich the diversity of perspectives within the various farming communities to ensure all information provided by the participants reflected the actual problems within the farming community. Also, recruitment considered crop diversity, as different crops attract distinct pest pressures and pesticide regimens. Data collection occurred during the main farming season, when pesticide use was at its peak, allowing the capture of practices under real working conditions.

Although the sample size (n = 13) is small compared to quantitative studies, it is consistent with qualitative research standards where depth of understanding is prioritized over numerical representativeness. Farmers were sampled purposively using criterion-based selection. Agricultural extension officers in the Hohoe municipality assisted the research team in identifying farmers who met the inclusion criteria . A list of eligible farmers was generated through community registers and local farming cooperatives. From this list, the research team approached potential participants in person at their farms, explained the study, and invited them to participate. Those who agreed provided informed consent before being enrolled. Sampling was iterative, with participants selected to reflect variation in age, gender, household size, and farming experience. Sampling continued until theoretical saturation was reached [17]. The theoretical saturation was determined when additional interviews yielded no new codes or themes relevant to the research questions. After the eleventh interview, only repetitive responses were noted; by the thirteenth interview, no novel insights emerged, confirming that saturation had been reached. The depth of ethnographic interviewing and attainment of saturation ensured that the data were sufficient to answer the study objectives.

### Data collection and analysis

The researcher conducted an in-depth interview. Consent was sought and obtained from each interviewee. All interviews were audio‐recorded, and anonymized transcripts were used for analysis. The topic guide was developed after reviewing prior studies on farmers’ pesticide knowledge, safety practices, and occupational risks [5,13,21].

Open‐ended questioning was employed, and the flow of each interview determined the order of questioning. The interview guide and subsequent thematic analysis were organized around four main domains: (1) knowledge and practices of pesticide use (types, reasons for use, and dosage determination); (2) health risks and exposure (self-reported symptoms and perceived causes); (3) safety practices (use of personal protective equipment, pre- and post-exposure management); and (4) pesticide storage and disposal (methods, locations, and environmental implications). Each domain included open-ended variables that allowed farmers to narrate personal experiences and perceptions related to occupational exposure.

Each interview began with a description of the participant’s farming experiences and sociodemographic characteristics. The interview guide was piloted with two farmers outside the study sample to ensure clarity, cultural appropriateness, and validity; minor revisions were made before full data collection.

Data collection was conducted between 13^th^ August and 14^th^ October 2024, coinciding with the peak pesticide application season for maize and vegetable cultivation in the municipality. This timing allowed the capture of farmers’ practices during periods of intensive pesticide use, thereby enriching the contextual validity of the findings.

Investigators familiarized themselves with the transcript data through repeated reading and reflection and independently subjected the transcripts to a process of open coding by going through the transcripts line by line. ATLAS.ti (Version 25.0.0) was used to apply thematic coding. This software was selected because it provides efficient management of large qualitative datasets, facilitates systematic coding, and supports retrieval of co-occurring themes. To enhance consistency, two researchers independently coded a subset of transcripts and compared results. Inter-coder reliability was established through iterative discussions until consensus was achieved, and a unified coding framework was applied to the remaining transcripts. The process of selective coding was used to generate related themes to explain the information of the participants.

### Ethical issues

Ethical approval for this study was sought from the University of Health and Allied Sciences Research Ethics Committee (UHAS-REC B.10[160]24–25) and written approval from the Municipal Health Directorate. Importantly, participants were allowed to give written consent by indicating their signature or thumbprint. During transcription, each participant was assigned an identification code (e.g., “Farmer 01,” “Farmer 02”) that replaced personal identifiers such as names, community, or farm location. The transcripts and audio files were stored separately on password-protected drives, with only the principal investigator having access. This ensured complete anonymity and confidentiality throughout the analysis process. All data obtained was handled confidentially. 

## Results and findings

**Fig 1:**
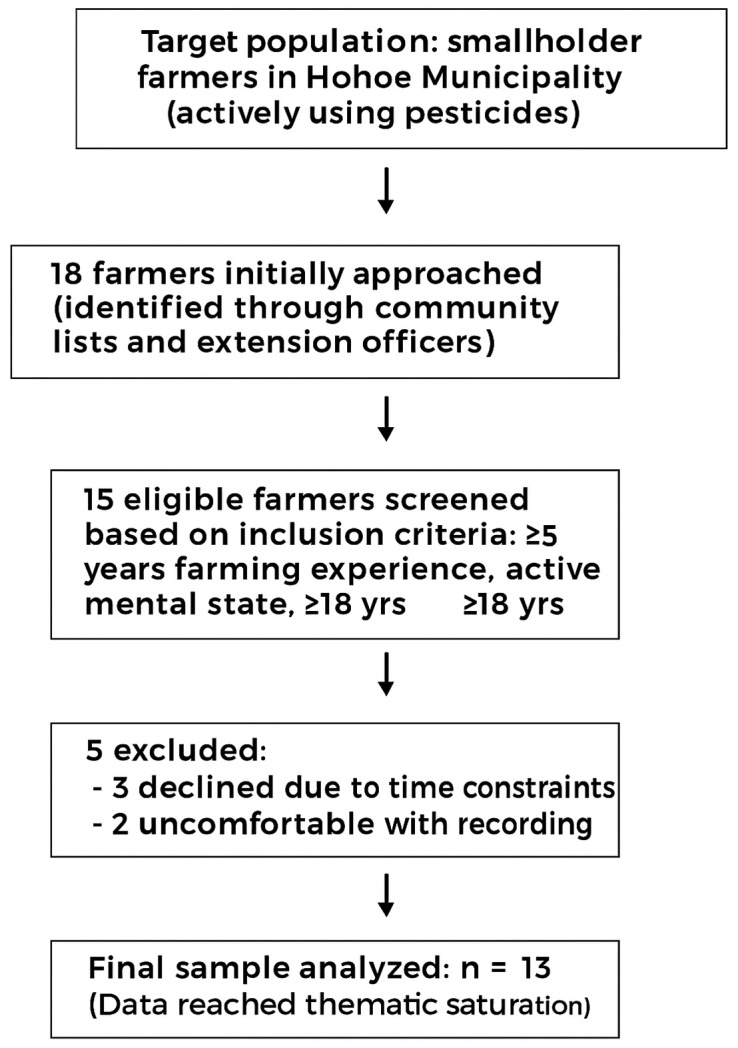
Flowchart illustrating participant recruitment and selection process for the qualitative ethnographic study on occupational pesticide exposure among farmers in Hohoe Municipality (n = 13).

A total of 18 farmers were approached for participation, of whom 13 consented and completed the interviews, giving a response rate of 72%. Five farmers declined due to time constraints or discomfort with audio recording. Fig 1 presents the flow of participant selection from the target population to the final sample.

The farmers were predominantly smallholder crop producers cultivating maize, cassava, okra, plantain, and vegetables such as garden eggs and peppers. Farm sizes ranged from approximately 0.5 to 3 acres, with the majority (8 of 13) managing family-owned plots rather than leased land. Seniority in farming ranged from 7 to 30 years, with an average of 15 years. Most farmers (10 of 13) used pesticides throughout their farming careers, often beginning within the first few years of practice when pest infestation became recurrent.

### Socio-demographic characteristics of the farmers

The socio-demographic characteristics of participants are summarized in Table 1 below. The majority (38.5%) of the farmers were between 30–39, while the 20–29 and 40 + age groups were equally lower (30.8%) each. Females (61.5%) were more involved in farming than males (38.5%). Most participants (69.2%) lived in households with fewer than 9 members, while a smaller proportion (30.8%) had 10–19 members. The majority of the farmers (38.5%) had completed Senior High School (SHS), while Junior High School (JHS/JSS) and Tertiary education had the lowest representation (15.4%) each. In terms of farming experience, the largest group (38.5%) had 10–19 years of experience, whereas the least experienced group (less than 9 years) accounted for 15.4 percent.

**Table 1:** Socio-demographic characteristics of the farmers.

Variable	Frequency(n = 13)	Percentage (%)
Age		
20-29	4	30.8
30-39	5	38.5
40+	4	30.8
Gender		
Male	5	38.5
Female	8	61.5
Household Size		0.0
Less than 9	9	69.2
10-19	4	30.8
Level of education		
Primary	4	30.8
JHS/JSS	2	15.4
SHS	5	38.5
Tertiary	2	15.4
Years of farming Experience (years)		
Less than 9	2	15.4
10-19	5	38.5
20-29	3	23.1
30 and above	3	23.1

### Thematic findings

Table 2 presents the themes of pesticide use among farmers for analysis. The main themes were knowledge and practices of using pesticides, health risks and exposure, safety practices, and storage and disposal of pesticides.

**Table 2:** Thematic table.

Main theme	Sub-theme	Codes
Knowledge and Practices of Pesticide Use	Type of pesticide use	• Glyphosate
		• Paraquat Dichloride
		• Gamma
		• 2, 4-D
		• Keystone
		• “Japata” (glyphosate-based herbicide)
	Reason for pesticide use	• Prevent insect infestation
		• Vendor recommendation
		• crop safety
		• Enhance crop growth
	Determining the quantity of pesticide	• Read product label
		• Use a calibrated measuring cup
		• Container (milk can, tin tomatoes)
		• Vendor advice
	Awareness of Expiry date	• Yes
		• No
	Application methods and frequency	• Spraying
		• Frequency varies (monthly, yearly, weather dependence)
Health Risk and Exposure	Health issues	• Eye irritation
		• Skin irritation
		• Waist pains
		• Loss of life and sight
Safety Practices	Safety material used (PPE)	• Boots
		• Overall (long sleeves, trousers)
		• Nose mask
		• Gloves
	Safe handling and application practices	• Use protective equipment
		• Handle pesticides with care
		• Partially fill the spraying machine
		• Walk backwards when spraying
	Post-exposure management	• Immediate rinse with water
		• Medication (neem tree)
		• Wash with soap
Storage and Disposal of Pesticides	Storage practices	• House (Storerooms, under bed)
		• Carton
		• Farm
	Disposal methods	• Discarded on the farm
		• Burn

Of the 13 farmers interviewed, 10 (77%) reported using boots, 7 (54%) wore long-sleeved shirts or trousers, 5 (38%) used gloves, 4 (31%) used nose masks, and only 2 (15%) mentioned goggles or face shields. None reported full PPE compliance during spraying.

### Knowledge and practices of pesticide use

In the findings, five (5) sub-themes emerged: type of pesticide use, the reason for pesticide use, determining the quantity of pesticide, awareness of expiry date, and application. These sub-themes give in-depth knowledge of pesticide use. Farmers explained that agrochemical vendors were the main sources of pesticides and advice. Vendors generally did not visit farms; rather, farmers purchased pesticides directly from shops or stalls in town markets. Only a few vendors occasionally toured farming communities during peak planting seasons. Most vendors had limited or no formal agricultural training; some had only received short informal sessions from pesticide distributors. As a result, farmers heavily relied on vendor recommendations when selecting and mixing/loading pesticides, increasing the risk of misinformation and unsafe application practices.

Regarding the type of pesticide use, the farmers identified various pesticides they commonly apply to their farms. They described these pesticides as essential for controlling weeds and pests that threaten crop yield. Specifically, they mentioned Glyphosate (gramazole), Paraquat Dichloride, glyphosate-based herbicide (Jupata), Gamma, and 2,4-D, Keystone, each serving different functions in their agricultural practices. Participants reported that,

*“Okay, we sometimes use King Kong; they said ‘jupata’ and ‘gramazole”* (Male, 21 years)

*“First, I used to use glyphosate. Glyphosate is the one I used to use, but at some point, I didn’t get the glyphosate I used, now “Edwoji” and King Kong are the ones I use so far”* (Male, 40 years)

Concerning the reasons for pesticide use, the farmers highlighted several motivations for incorporating pesticides into their farming practices. They emphasized that the primary reason was preventing insect infestation, as they explained that without pesticide application, crops would be highly vulnerable to pest attacks, leading to significant yield losses. Additionally, some participants noted that their choice of pesticides was based on vendor recommendations; thus, they rely on agrochemical sellers for guidance on which pesticides to use. This suggests that vendor influence plays a crucial role in shaping farmers’ pesticide selection and usage patterns. They also stated crop safety, as farmers explained that pesticides help protect crops from insects. They believed that without pesticides, their crops would be weaker and more susceptible to damage, ultimately affecting their food supply and income. Farmers commonly expressed that,

*“Ummmh,” the okro, if you leave it and you didn’t use that medicine, the insect will get into it and it will spoil”* (Female, 32 years)

*“I ask the seller what to use for my crops, and follow their advice” (Male, 40 years)*

Interestingly, several farmers referred to pesticides as “medicine” for crops, reflecting a cultural perception that equates chemical treatment with healing or strengthening plants rather than recognizing their toxic properties. This symbolic view is explored further in the Discussion.

With the determination of pesticide quantity, they have employed various methods to measure the appropriate amount for application. One common approach was reading the product label, as some farmers explained that they followed the manufacturer’s instructions to ensure accurate pesticide dosage. However, not all farmers strictly relied on product labels. Some mentioned the use of a calibrated measuring cup, which provided a standardized way to measure pesticide quantities. This method was perceived as more precise, especially among those who had access to proper measuring tools. Others, however, adopted more informal measurement techniques, such as using household containers like milk cans or tin tomato cans. This method was based on their experience as farmers. They also indicated that in some cases, vendor advice influenced the quantity of pesticide they applied. This is expressed by some farmers that;

*“Sometimes it’s written on the container, so per the litter, sometimes they can say 500ml per litter”* (Female, 29 years)

*“First, I used to ask the seller because mostly they also buy it somewhere. So, they ask the one they buy it from, so as they also know a little about it, they also advise me that I have to apply this quantity to the water, before I spray. That’s exactly what I do”* (Male, 24 years)

About awareness of pesticide expiry dates, the farmers exhibited varying levels of knowledge and practices. Some farmers acknowledged that they check expiry dates before purchasing and using pesticides; they stated that expired products might lose effectiveness or pose risks to the crop. They explained that ensuring the pesticide is within its valid period helps maintain its intended potency and prevents unintended harm to their crops. However, other farmers admitted that they do not check expiry dates when purchasing pesticides. They explained that their focus is primarily on the pesticide’s function rather than its expiration status. Some assumed that if the pesticide is available for sale, it must still be effective. Most older participants did not check expiry dates, while younger farmers were likely to mention expiry checks. Farmers who ignored expiry dates often assumed that products sold in shops must still be effective. Illustrative quotes included,

*“I have to check because if I didn’t check, I would end up buying something that will not work out on my farm for me” (Male, 40 years)*

*“Hahaha, oh no, i dont check expiry date because I don’t know anything about ” *(Female, 66 years)

Concerning application methods and frequency, the study identified spraying as the most common. All farmers used knapsack sprayers operated manually by pumps; none reported using motorized or tractor-mounted sprayers. Most Knapsacks were older than three years, frequently borrowed or shared among farmers, and rarely serviced. Worn nozzles and leaking hoses were a common sight, increasing the likelihood of direct dermal exposure. Only one farmer reported regular maintenance, including rinsing and checking seals before each spraying season.

The farmers explained that spraying allows for even distribution of the pesticide across crops, ensuring effective pest and weed control. All participants reported beginning pesticide use within the first few years of starting farming, often after experiencing severe pest infestations. Most farmers (10 of 13) had used pesticides continuously since then. A few older farmers (3 of 13) indicated they had changed brands or reduced frequency in response to health symptoms such as dizziness or eye irritation, but none had stopped using pesticides entirely. None of the farmers received formal training before their first use. In terms of frequency, pesticide application varied among the farmers. Some reported applying pesticides every month, particularly for crops that require regular pest control to maintain their health and yield. Others indicated that they used pesticides seasonally or yearly, depending on the crop type and level of infestation. Additionally, a few farmers mentioned that weather conditions influenced their pesticide application schedules. For instance, heavy rains could wash away pesticides, requiring reapplication, while prolonged dry conditions might reduce the need for frequent pesticide use. These variations in application methods and frequency highlight the adaptive nature of pesticide use among farmers. The findings also suggest that while spraying is widely accepted, there is no standardized approach to pesticide application frequency, leading to potential differences in effectiveness and safety.

*“No, for the spraying we use the machine”* (Female, 66 years)

*I use them depending on the weather, because some can be used and the rain washes them away, so I always study the season and the wind that blows”* (Male, 59 years)

### Health risks and exposures

Regarding health issues associated with pesticide use, the farmers reported experiencing various health effects, ranging from mild irritations to severe consequences. They mentioned eye irritation, which some farmers attributed to direct exposure to pesticide sprays during application. They also reported skin irritation, describing symptoms such as itching, rashes, and burning sensations after coming into contact with pesticides. Additionally, some farmers also complained of waist pains, which they linked to the physical strain of carrying pesticide-spraying machines and repetitive bending during application. They also stated severe health effects, including cases of vision loss and reported deaths among farmers who were heavily exposed to pesticides. These cases highlight the potential dangers of inadequate pesticide safety practices and prolonged exposure to toxic chemicals. Farmers reported that,

*“It itches me anytime I use it, and it’s very strong and highly scented.” *(Male, 30 years)

*“Okay, that one (Glyphosate), it has passed through the eye glass and then entered into my face before, let me say. Okay, so I feel like it’s harmful to my eyes.”* (Male, 40 years)

### Safety practices

Under this main theme, three (3) main sub-themes emerged that explain the safety procedures the farmers employed in applying pesticides. These themes were safety material use (PPE), Safe handling and application practices, and post-exposure management. Concerning the safety material used in pesticide application, farmers commonly counted their ordinary long-sleeved shirts and trousers as protective wear, equating regular clothing with PPE. Only a few used industrial coveralls. They mentioned boots, which were the most PPE they used. They also used coveralls, which included long sleeves and trousers, nose masks, and gloves. After spraying, most farmers stored their work clothes and PPE in the same rooms where they kept pesticides, often under beds or in storerooms, posing a risk of secondary contamination to household members. None reported dedicated cleaning or drying areas for PPE. Some washed their spraying clothes with regular household laundry, while others reused unwashed garments for several applications. Cleaning of sprayers was inconsistent; only a few farmers (3 of 13) rinsed equipment after use, while most left residues inside the tanks until the next spraying cycle. They emphasized that this protective equipment protects them from direct exposure to the pesticide. Participants reported that,

*“Yes, we wear long sleeves, boots, and socks.”* (female, 66 years)

*“I wear overalls, nose mask, and gloves.”* (Male, 19 years)

Regarding safe handling and application practices, the farmers described various precautionary measures taken to minimize the risks associated with pesticide application. These strategies were aimed at reducing direct contact with pesticides, preventing accidental exposure, and ensuring safe handling practices. Most farmers reported partial PPE usage, especially boots and gloves, while eye protection was consistently neglected. Younger farmers often mentioned gloves or masks, whereas older farmers relied on improvised clothing, such as long sleeves or pantyhose.

In addition to PPE, the farmers emphasized the importance of handling pesticides with care. This involved carefully mixing/loading and applying chemicals, avoiding spills, and ensuring that pesticide containers were properly sealed and stored. Some farmers also noted that reading product labels and following dosage instructions were essential in preventing excessive exposure. They also reported the practice of partially filling the spraying machine instead of completely loading it. Farmers explained that overfilling the sprayer increased the risk of leakage and spills. Additionally, they also reported adopting the practice of walking backward while spraying to avoid stepping on freshly treated areas and minimize direct contact with pesticide residues. Some farmers reported that:

*“Okay, if you are spraying, you have to be spraying and walking backward and not spraying forward”* (Female, 32 years).

*“So I wear my pantyhose before, wearing multiple layers of clothing and sometimes farm boots as protective measures*” (Female, 38 years)

Concerning post-exposure management, participants described various actions taken to mitigate the effects of accidental pesticide exposure. Immediate rinsing with water emerged as the common safe handling and application practice. Farmers explained that when pesticides accidentally spilt on their skin, they quickly washed the affected area with large amounts of water to minimize absorption and irritation. This practice was recognized as an essential first-aid measure, though some participants admitted they did not always have immediate access to clean water in the field. They also reported the use of medicinal plants, particularly the neem tree, as a medication when exposed to pesticides. They also stated that they washed with soap after pesticide exposure to remove any chemical residues left on their skin or clothing. This practice was considered effective in reducing prolonged contact with pesticides and preventing further irritation. Illustrative quotes included,

*“If it comes like that, I will use the ‘neem tree’, uh-huh. That same day when it happened, I use the ‘neem tree’, I cooked it, and I drink it.”* (Female, 32 years)

*“I’m aware that it hurts, so immediately it pours; I rinse with plenty of water.”* (Female, 38 years).

### Storage and disposal of pesticides

Under this theme, the farmers explain how they store the remaining pesticide and the various ways of discarding it.

For storage practices, the farmers reported different locations for storing their pesticides. Most of them stated they stored it inside their homes, particularly in storerooms or under beds. This was due to concerns about theft, as some farmers feared that leaving pesticides on the farm would result in them being stolen. Others preferred to store them in cartons, possibly to keep them organized and prevent spills. Some also reported storing them on their farms. Some farmers reported that:

*“I put it in the room, but not in our bedroom, the store room”* (Female, 38 years)

*“I put it in the carton, and then I hide it under my storeroom”* (Male, 40 years)

With disposal methods, farmers left empty pesticide containers on their farms, either discarding them openly or burying them. This raises concerns about soil contamination and long-term environmental hazards. Some farmers also burnt pesticide containers, which, while reducing waste, could release harmful fumes into the air, posing health risks. However, these practices contradict Ghana EPA guidelines, which prohibit open burning and encourage safe collection and disposal. Farmers’ limited awareness of these rules highlights a gap between policy and practice, emphasizing the need for education on environmentally friendly disposal techniques. Some farmers reported that:

*“Okay, sometimes, we keep it in the farm at one place, yeah”* (Male, 21 years)

*“For the containers I used to pack them. And sometimes we burn them also”* (Female, 29 years)

## Discussion

This study explored occupational pesticide exposure, safety practices, and perceptions among smallholder farmers in the Hohoe Municipality of Ghana. The findings revealed widespread pesticide use, primarily Glyphosate, Paraquat Dichloride, Gamma, 2, 4-D, Keystone, and “Japata” (glyphosate-based herbicide), with most farmers relying on vendor advice for selection and dosage. Health complaints such as eye and skin irritation, waist pain, and vision problems were common. While some farmers used partial PPE, none demonstrated full compliance. Unsafe storage and disposal practices, including keeping pesticides under beds or burning empty containers, were prevalent. The study also revealed that farmers perceive pesticides as “medicine” for crops, reflecting cultural understandings that may influence risk perception and behaviour.

The inadequacy of farmers’ knowledge regarding pesticide safety raises major public health concerns. Indeed, smallholder farmers in Hohoe are not fully aware of the dangers posed by the chemicals based on their pesticide application practices. This lack of awareness can lead to accidents, prolonged exposures, and contamination, not only for the agricultural workers themselves but also for consumers and the environment. Consistent with this study, a study conducted among small-scale farmers in Ethiopia revealed that 95% of farmers had poor knowledge regarding pesticides [22]. However, another study revealed that 92.4% of farmers had adequate knowledge of the use of pesticides [23]. The divergence between Ethiopia [22], where knowledge was poor, and Nepal [24], where knowledge was higher, may reflect contextual differences. Ethiopia has weaker pesticide regulation and lower farmer-to-extension officer ratios, limiting formal safety training. Nepal, by contrast, has introduced structured pesticide awareness campaigns and predominantly cultivates crops (e.g., rice, vegetables) with higher pesticide regulation, which may increase farmer familiarity with safe practices. Such contextual differences suggest Ghana’s challenges may align more closely with Ethiopia, given weak regulatory enforcement and dependence on informal vendor guidance.

[21] established a strong relationship between knowledge of pesticide use and actual pesticide application practices. In line with this, the current study found a poor practice of pesticide use among farmers, which exposes them to various health issues.

Poor pesticide use and exposure among farmers pose severe health risks due to unsafe practices, including improper storage, inadequate PPE use, and hazardous disposal [25]. Consistent with studies in Ghana and globally, farmers in Hohoe reported a combination of acute symptoms such as headaches, dizziness, and skin/eye irritation, along with longer-term complaints like respiratory problems and musculoskeletal pain, impotence and chronic cough. Although prevalence rates vary [26,27], the pattern is clear: inadequate PPE and unsafe handling expose farmers to both immediate and chronic health risks.

These health issues not only endanger the health of farmers but also perpetuate cycles of poverty and environmental degradation. According to a study by [28], just 35% of farmers wear complete Personal Protective Equipment (PPE) when applying pesticides, while 45% wear partial PPE, which includes some or all of the following: a respirator, goggles, cap or headgear, rubber gloves, dungarees, and Wellington boots. However, 20% of farmers in the research region used pesticides without donning Personal Protective Equipment. These practices predispose farmers to both acute toxicity and chronic health issues. Although wearing PPE in part (such as boots and gloves) lowers certain exposure risks, there are still important gaps that expose farmers to direct chemical contact, such as failing to wear eye protection. For example, fumes or splashes from herbicides like glyphosate and Paraquat Dichloride can permanently harm ocular tissues. [29] found that the lack of goggles was associated with eye discomfort and cases of vision loss. In a similar vein, irregular nasal mask use increases the risk of inhalation and contributes to respiratory problems, such as dizziness and persistent cough [30]. An essential component of using pesticides responsibly is disposing of the residue from them properly. People can be harmed and the environment contaminated by the unintentional or uncontrolled emission of pesticide waste. In this study, some participants exhibited unhealthy practices related to the storage and disposal of pesticides. According to [31], inadequate pesticide storage was demonstrated by the disparity in storage facilities; the utilization of empty pesticide containers for household purposes was determined to be 20.2%.

The study’s focused ethnographic design allowed for an in-depth understanding of farmers’ lived experiences, providing rich contextual insights that would be difficult to capture through surveys alone. Triangulation of interviews and field observations enhanced the credibility of findings.

However, several methodological limitations may have introduced bias. The purposive selection of farmers with more than five years of experience may have excluded newer or seasonal farmers who could display different levels of awareness or risk behaviours. This selection bias limits variability but enhances internal consistency, as experienced farmers are more likely to have sustained pesticide exposure and established routines. Additionally, the small sample size (n = 13) limits representativeness, although thematic saturation was achieved.

Similarly, reliance on self-reported data could have resulted in recall bias or social desirability bias, as farmers may underreport unsafe practices. The absence of direct observational or biomarker data prevents quantification of exposure. Despite these limitations, rigorous interviewer training, standardized topic guides, and cross-checking of coded transcripts strengthen the study’s validity.

While the results are specific to the Hohoe Municipality, they echo findings from similar rural agricultural settings across sub-Saharan Africa. For instance, a cross-sectional study in Ethiopia among small-scale vegetable farmers found that 95% of participants had poor knowledge of pesticide safety and limited PPE use [22]. Similarly, a study in Tanzania reported frequent paraquat exposure and inadequate protective behaviour among cotton farmers, leading to acute poisoning cases [10]. The consistency of these findings across diverse African contexts underscores the generalizability of the occupational health challenges observed here, though local differences in pesticide regulation and training opportunities may influence severity and outcomes.

However, the unique cultural framing of pesticides as “crop medicine” in Hohoe may limit direct comparability to non-Ghanaian contexts, emphasizing the need for locally adapted interventions that respect community beliefs and practices.

The results highlight that interventions should move beyond individual-level training and PPE provision. Although improving farmers’ knowledge and encouraging PPE use remain critical, these measures alone cannot eliminate risk. Broader structural and regulatory measures, including stronger enforcement of pesticide registration laws, monitoring of agrochemical vendors, and banning of highly hazardous formulations, are essential to ensure safer agricultural practices.

Legislation should mandate vendor certification and require that only approved, well-labelled pesticides are on the market. Moreover, occupational safety education should be integrated into Ghana's agricultural extension programs, ensuring periodic refresher sessions for farmers.

From a public health perspective, PPE should be considered the last line of defence, not the primary safeguard. Emphasis should instead be placed on upstream controls, such as safer pesticide formulations, proper storage facilities, and clear disposal systems, to reduce exposure at the source. Establishing community-level collection points for empty containers and linking farmers with local health units for medical surveillance would further strengthen occupational safety.

This study provides important qualitative evidence of unsafe pesticide practices and associated health risks among smallholder farmers in Hohoe. Despite awareness of pesticide toxicity, socioeconomic constraints and weak regulatory oversight perpetuate unsafe behaviours. Strengthening legislation, vendor regulation, and agricultural extension services, while ensuring that safer products reach farmers, is crucial. Sustainable reduction in pesticide-related illnesses will depend not only on behaviour change but also on structural reforms that protect farmers, their families, and the environment.

### Recommendations

Based on the findings of this study, several context-specific interventions are proposed to improve pesticide safety among farmers in the Hohoe municipality. The Ministry of Food and Agriculture, in collaboration with the Ghana Environmental Protection Agency (EPA), should institute regular farmer training programs focused on correct pesticide dosage, consistent use of complete Personal Protective Equipment (PPE), especially goggles and respirators, and safe disposal of pesticide containers. These trainings should be delivered in local languages, use practical demonstrations, and be tailored to the literacy levels of farmers to ensure comprehension and adoption.

Furthermore, agrochemical vendors, who are a primary source of advice for farmers, should be trained, certified, and closely monitored to ensure they provide accurate, evidence-based information on pesticide application. Vendor regulation would reduce misinformation and the unsafe recommendations that currently shape farmers’ practices.

Moreover, local assemblies should establish community collection points for empty pesticide containers and enforce Ghana EPA regulations that prohibit unsafe practices such as burning or discarding containers on farms. Public awareness campaigns in farming communities can further reinforce safe disposal practices and reduce environmental contamination.

Finally, interventions should address socio-cultural and economic realities in Hohoe. This includes engaging both male and female farmers in training programs, providing subsidized PPE to overcome cost barriers, and strengthening links with local health facilities for routine health monitoring of farmers exposed to pesticides.

## Conclusion

This study highlights critical gaps in knowledge, practices, and safety measures related to pesticide use among smallholder farmers in the Hohoe municipality. Despite their central role in sustaining local food production, many farmers continue to apply pesticides without complete protective equipment, often store agrochemicals unsafely within households, and dispose of containers in ways that endanger both health and the environment. These practices contribute to self-reported health problems such as eye and skin irritation, respiratory discomfort, and, in severe cases, vision loss and deaths.

The findings underscore that improving pesticide safety cannot rely solely on farmer awareness but requires stronger occupational health policy integration and effective local enforcement. Policies on pesticide regulation and safe disposal must be enforced at the community level, supported by agricultural extension services and local assemblies. At the same time, equipping farmers with affordable PPE and practical, culturally appropriate training can reduce their vulnerability to both acute and chronic pesticide-related illnesses.

Ultimately, protecting farmers’ health and livelihoods in Hohoe is not only a public health priority but also a necessary step toward sustainable agriculture and food security in Ghana. Ensuring that occupational health safeguards are embedded into agricultural policy and enforced at the grassroots level will be key to achieving this goal.
